# Implementation of Activity-Based Workplaces (ABW)—The Importance of Participation in Process Activities

**DOI:** 10.3390/ijerph192114338

**Published:** 2022-11-02

**Authors:** Eva L. Bergsten, Katarina Wijk, David M. Hallman

**Affiliations:** 1 Department of Occupational Health Sciences and Psychology, Faculty of Health and Occupational Studies, University of Gavle, 80176 Gavle, Sweden; 2 Centre for Research and Development, Uppsala University, Region Gavleborg, 80187 Gavle, Sweden; 3 Department of Public Health and Caring Sciences, Uppsala University, 75123 Uppsala, Sweden

**Keywords:** activity-based flexible office, office design, relocation, organizational intervention

## Abstract

Relocation to new office solutions such as activity-based workplaces (ABW) has increased but satisfaction with the ABW among employees varies, and the importance of participation in the relocation process is unclear. This study aimed to examine the association between employees’ extent of participation in the implementation process activities and satisfaction with the relocation to ABW. Data were collected from 699 employees in a Swedish governmental agency 3-months prior to, 3-months and 9-months after relocation to the ABW. Questionnaires were used to assess participation in process activities and perceived satisfaction with knowledge about working in ABW, office rules, and information and support during the process. Participation in activities was significantly associated with higher overall satisfaction with knowledge, office rules, information and support, and effects were generally more pronounced as the number of attended activities increased. Satisfaction also increased among non-participants, although without reaching the same levels as participants. Our results show the importance to offer and facilitate a high participation in the relocation process activities to obtain satisfaction with a relocation to ABW.

## 1. Introduction

There is an increasing interest in creating new office solutions, and in particular activity-based workplaces (ABW). ABW can be defined as open-plan offices with unassigned and shared desks, in workspaces that aim to enable and support employees different work behaviors, and activities requiring, e.g., high concentration, confidentiality, communication, collaboration and interaction with others [1,2]. Together with the digitalization office work has become more flexible in where to work. With a decreased office occupancy, organizations can save costs with an ABW design through a more efficient use of office space [2,3,4,5]. Organizations also believe, and studies have shown, ABWs to increase collaboration and interaction among employees [5,6], which emphasize a need to design and implement satisfying ABWs that enhance interaction and ad hoc meetings.

However, successful implementations of ABW requires that employees understand the concept [1,5,7], including what characterizes the ABW, for example office rules [8] and behaviors [9] that facilitate an activity-based way of working. Surprisingly, little attention has been paid to the implementation process and activities influence on satisfaction with and adoption to the ABW [7,10,11,12]. A qualitative study by Brunia & De Been (2016) compared successful and unsuccessful implementations of ABWs and found that, sound information and communication, management commitment, opportunities for employees to discuss the ABW concept and quick response to questions, were important factors for satisfaction with the ABW. In line with these results, another qualitative study showed that addressing the ABW concept during the process increased knowledge and acceptance of the concept, and thus, prepared employees for how to use the ABW [8]. Discussing and deciding on office rules in the planning and implementation process also decreased negative feedback on the work environment. A review by Marzban et al. (2021) suggested that the shortcomings of ABW were related to the implementation of a new way of working rather than the office concept [13]. Research evaluating the implementation process of ABW is still limited, and most of the previous studies used qualitative methods. This study will examine the importance of the process on knowledge, office rules and information and support over time, by use of quantitative measures.

Participation in implementation of interventions is associated with the perception of improved working conditions in implementation research [14,15]. More specifically, the relocation process has impact on employees reactions to change, new ways of working, productivity and employee satisfaction which partly derive from the relocation process [16]. Relocation affects employees differently and evoke different expectations and needs both during the process and after relocation. This needs to be understood and considered by the companies and employees needs to be guided and supported through the relocation process [17]. Interventions are not always based on theories but needs to reflect on the assumptions about what will produce change [18]. In this study, we systematically followed the natural implementation of an ABW and evaluated the company objectives with the implementation process, which was to increase *knowledge* about ABW, to understand *office rules*, and to administer a transparent process with satisfying *information* and *support*. The implementation process relied on participatory ergonomics approach intended to involve employees in ergonomic analysis and design and are commonly used to develop skills, activities and competencies [19]. The process included four optional process activities, ergonomic seminar, management information, workshops and an inspiration seminar. The process aimed to prepare employees for the relocation to ABW and adopt a new activity-based way of working.

The aim of this prospective study is to examine the association between participation in the implementation process activities and employees’ satisfaction with *knowledge* about working in ABW, *office rules, information* and *support* during the process, before and after the relocation to ABW. Our hypothesis is that participation in process activities is associated with satisfaction with the relocation to ABW, and that satisfaction increase with the number of activities attended.

## 2. Methods

### 2.1. Background

We followed the relocation from traditional offices offering cell-offices and open-plan landscapes to ABW in two office sites within a large government agency in Sweden. The relocations took place in August 2018 and January 2019, while the implementation of activities to prepare employees for a new way of working started in the beginning of 2018 for both office sites. Together with expert consultants the company planned and implemented activities to prepare employees for the new activity-based way of working. According to the organization, this implementation process was a consequence of previously, less successful, relocations to ABWs within the organization applying a less explicit implementation process. Based on that experience the organization highlighted four objectives to accomplish with this implementation process, to increase employees’ *knowledge* about ABW, facilitate understanding of the new *office rules*, and offer a transparent process by satisfying *information* and *support* to employees. The implementation process and tailored activities were planned and realized with no interference from the researchers, but in collaboration with expert organizations to operate some of the activities.

### 2.2. Design

This intervention study used a prospective design to evaluate changes in satisfaction during relocation to ABW in employees who participated in process activities compared with non-participants. After implementation of activities, questionnaire data were collected at three waves; 3-months prior to, 3-months and 9-months after relocation to the ABWs. All participants signed an informed consent prior to participation. The study was approved by the Regional Ethical Review Board in Uppsala, Sweden (Dnr.2015/118).

### 2.3. Implementation Activities

Prior to the relocations four major activities were offered to all the employees, *modern ergonomics seminar*, *company information*, *workshops*, and *inspiration seminars.* All the activities were voluntary and offered at the workplace during office hours. The *modern ergonomic seminar* was offered twice, to inspire an activity-based way of work and understand the ergonomic benefits with mobility in the office and was given by an expert consultant. The *management information* activity was offered at three occasions, concerned the local implementation of ABW, why and how to implement the ABW, with the possibility to ask and discuss questions, and was held by the project management. *Workshops* were offered at nine occasions and aimed to give knowledge and tools for how to utilize activity-based work. The *inspiration seminar* was offered at three occasions concerning changes in today’s working life, how to work activity based, why and how agencies implement ABW, threats and expectations and, being a manager in ABWs. The seminar was given by expert consultants.

### 2.4. Data Collection

#### 2.4.1. Participants

All eligible employees (*n* = 1061), at the two-office sites, were invited to participate in the study via questionnaire. Employees who did not move to ABW, were on sick leave, parental leave, reporting job changes or retirement in advance, or were about to receive prioritized seats in the ABW, were defined as not eligible and did not receive the questionnaire. Inclusion required response at baseline and at least one of the two follow-ups. The participants and data collection have been described in detail elsewhere [20].

#### 2.4.2. Questionnaire

A web-based questionnaire with a personal link was sent to all eligible employees by e-mail, followed by three weekly reminders. The questionnaire contained questions about age (years), gender (man, woman, do not want to categorize), office type (cell-office, shared room (2–4 persons), open-plan office (>4 persons), ABW), position (manager or employee), participation in process activities (yes or no) and different aspects of satisfaction. Employees were divided in participant and non-participant groups. Participation (yes or no) and the number of attended activities (0 to 4) were used as independent variables.

Dependent variables were perceived satisfaction with *knowledge*, *office rules*, *information*, and *support* [20]. Four questions were developed based on interviews with employees and customized to match the four company objectives regarding the relocation process. Questions were asked as “to what extent do you...” “receive the knowledge you need about ABW to feel confident?” (*knowledge*), “begin to understand the new office rules?” (*office rules*), “receive the information you need at the right point of time?” (*information*), and “know who to address your questions?” (*support*). Questions were rated on a six-point response scale from 1 (“not at all”) to 6 (“to a very large extent”). However, due to an error in the questionnaire system, the last follow-up measurement included seven response options, although the endpoints were the same. Thus, all responses were converted to s scale from 0–100 with higher values indicating more satisfaction.

The questions about age, sex and satisfaction with the psychosocial work environment, rated on a five-point scale from “not at all” to “very” satisfied, were used as confounders.

### 2.5. Statistical Analyses

Statistical analyses were performed using IBM SPSS Statistics 24 (Armonk, NY: IBM Corp). Descriptive statistics are presented as means and standard deviations (SD), frequencies and percentages. Differences in baseline data between the participant and non-participant groups were determined using t-test for continuous variables and Chi^2^-tests for proportions. The effects of participation in activities on satisfaction with knowledge, office rules, information and support were determined using linear mixed models with restricted maximum likelihood estimation (REML) of variance. Missing data were considered as missing at random. Participants with data at baseline and at least one of the two follow-up measurements (three or nine months) were included in the analyses. The models for each outcome were constructed with *participation* (five levels: participating in 0–4 activities), *time* (three levels: baseline, 3-months and 9-months follow-up) and the interaction (*participation* × *time*) as fixed factors. Subject and intercept were included as random effects using variance components as the covariance structure. In addition, the models were adjusted for satisfaction with psychosocial work environment (rated on a five point scale from 1 “not at all” to 5 “very” satisfied), age (years) and sex (man or woman). In each model, we determined the estimate (B) with 95% confidence intervals (CI). *p*-values < 0.05 were considered as statistically significant.

## 3. Results

### 3.1. Descriptive Statistics

Of the 1061 invited employees, 699 employees answered the questionnaire during baseline and at least one of the two follow-ups and were therefore included in this study. Of the included sample 439 participated in one or more activity (participants) and 260 did not participate in any activity (non-participants).

Descriptive baseline data of the study sample are shown in Table 1 for the participant and non-participant groups. The two groups did not differ in age (*p* < 0.001) but the non-participants contained a larger proportion of men (*p* < 0.001). Private offices were more common in the participant group than in the non-participant group before relocation (*p* < 0.001).

**Figure 1:**
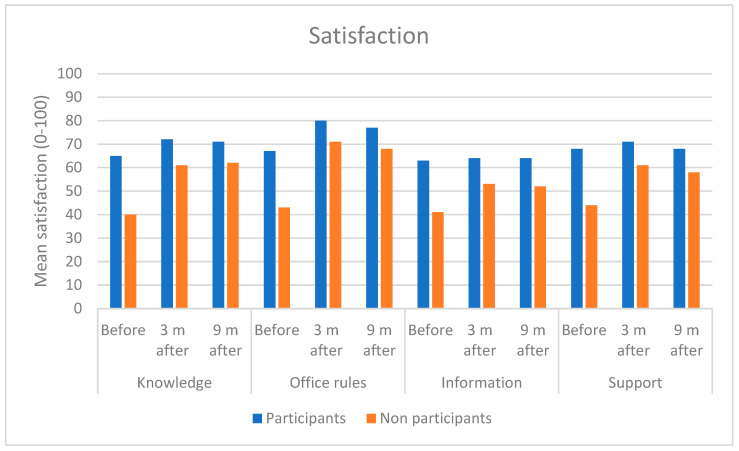
Mean values on a group level for satisfaction with knowledge, office rules, information and support in the participant and non-participant groups before, three and nine months after implementation of activity-based offices.

Perceived satisfaction with knowledge, office rules, information and support was rated higher in the participant group at baseline (*p* < 0.001) (Figure 1).

### 3.2. Changes in Satisfaction with Knowledge, Office Rules, Information and Support

Relocation to ABW was associated with improved satisfaction with knowledge, office rules, information and support. Satisfaction increased significantly three months after relocation compared to baseline (all outcomes *p* < 0.001) and the effects did not change markedly nine months after (Table 2). Adjusting for age, gender and satisfaction with the psychosocial work environment showed similar results, although stronger estimates for satisfaction with office rules (three months) and for knowledge and information (nine months) after relocation (Table 2).

### 3.3. Dose—Response Effects of Participation in Activities on Satisfaction with Knowledge, Office Rules, Information and Support

Participation in activities was significantly associated with higher overall satisfaction with knowledge, office rules, information and support (i.e., before, 3 and 9 months after relocation), and effects were generally more pronounced as the number of attended activities increased (Table 3). For example, attending 4 activities was associated with an increase in satisfaction with knowledge of 37 units (scale 0–100) compared with not attending any activity. Similar results were found when adjusting for age, gender and satisfaction with the psychosocial work environment, although the effects were somewhat smaller (Table 3).

### 3.4. Dose—Response Effects of Participation in Activities on Changes in Satisfaction after Relocation

**Figure 2:**
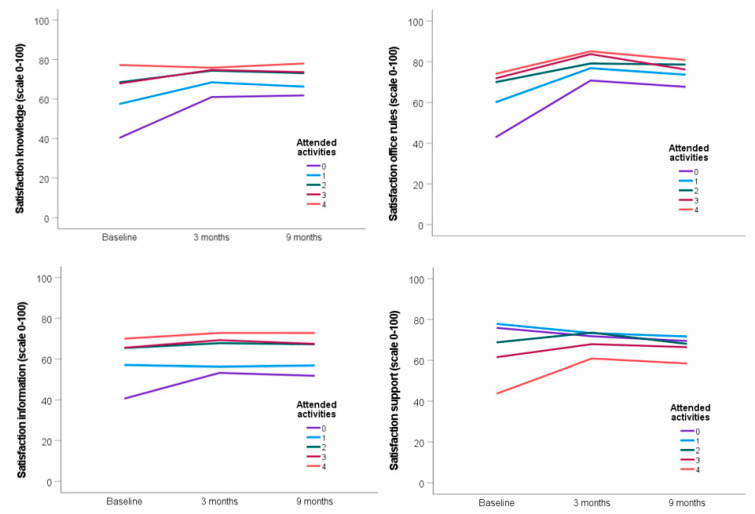
The figures show the change in rated satisfaction with knowledge about ABW, office rules, information and support at baseline, 3 and 9 months after relocation when participating in 1–4 activities, compared to the change in the non-participation group.

Table 4 shows the change from baseline (three months before) in satisfaction over time when participating in 1–4 activities, compared to the change in the non-participation group. The change in satisfaction with knowledge three and nine months after relocation was less pronounced among the participant groups compared with non-participants (i.e., negative estimates), although attending one activity was only significant after nine months. This effect was stronger for those attending more activities, as these groups reached higher satisfaction even before relocation and remained more satisfied after (see Figure 2). A similar pattern was found for satisfaction with office rules and support. However, the number of attended activities did not seem important for satisfaction with information (i.e., all but one estimate were non-significant).

## 4. Discussion

In this prospective study we aimed to understand the importance of office workers’ participation in the implementation process activities offered by the company during relocation from traditional to activity-based offices. Our hypothesis, that participation in process activities would affect and increase knowledge about working in ABW, office rules, and information and support during the process, was realized. Satisfaction with knowledge, office rules, information and support increased among participants at three months follow-up and for knowledge and information also after nine months after adjusting for age, sex and satisfaction with the psychosocial work environment. In addition, the effect estimates increased with the number of attended activities. Our results also showed an increase in satisfaction among non-participants, but on a lower level than participants.

### 4.1. Effects of Participation in Process Activities

Our results showed that satisfaction with knowledge about ABW, use of office rules, information and support during the process was increased shortly after relocation (three months follow up). The strongest short-term effect was satisfaction with office rules. Accordingly, Babapour et al. (2019) suggested that explicit and unambiguous office rules contribute to employee satisfaction with ABW, and accordingly rules are crucial for satisfaction with the working conditions in ABWs [8]. The understanding and benefits of using office rules seems to become clearer and may be more satisfying when practicing them in the office and therefore increased shortly after relocation.

Information and support during the implementation of ABW have previously been shown as important determinants of satisfaction with office relocation [11,17,20,21,22]. Our results confirmed that satisfaction with information increased with the extent of participation, i.e., the number of attended activities. In line with our results, employees’ perceived lack of opportunities to receive accurate information during the process contributed to negative experiences and a decrease in satisfaction with the environment in other office relocations [17,21]. It seems obvious that information and support needs to be prioritized in the implementation process to achieve employee satisfaction with the relocation. Overall, our results indicate that participation in more process activities generally associates with increased understanding of the ABW concept and support employees in the relocation. Thus, organizations should offer several activities (e.g., information, workshops, and seminars) to involve the employees early before relocation to new offices.

Employees that did not participate in any of the activities, non-participants, also increased satisfaction during follow-up, although they did not reach the same levels of satisfaction as the participants. Non-participants are of course not uninfluenced by the implementation process. However, participants most probably obtain more knowledge earlier in the process and therefore satisfaction may not increase to a marked extent after relocation. Thus, our findings suggest that facilitating the participation rate in process activities before relocation may accelerate the process of relocating to ABW.

### 4.2. Strength and Limitations

A strength with this empirical study is that we, in close collaboration with the organization, reached a large study population with a satisfying response rate. Further, we asked the employees very specifically about participation in each activity and not in general and, with two follow-ups which is more than in many studies within this field. This study also contributes with knowledge about the importance of employee participation in the implementation process of ABW, which is still a very limited research area.

Among limitations, data were self- reported which may be prone to different biases. Thus, future studies may attempt to collect more objective data on similar outcomes. Further, the questions used to measure satisfaction were not validated. However, they were developed specifically to match the four objectives, on the basis of dialogues with the organization, and in focus group interviews with employees [20], which may support their validity to measure these constructs. Another limitation is the potential for reversed causality because employees more satisfied with their work environment may tend to participate in the activities to a larger extent. Therefore, we adjusted for satisfaction with the psychosocial work environment and found no marked differences in results. Since the employees represented only one organization, caution is needed for generalization of the results to other organizations or work contexts.

## 5. Conclusions

Relocation to ABW effect employees differently and our results showed that satisfaction with knowledge, office rules, information and support increased among participants at follow-ups, and the effect estimates also increased with the number of attended activities. Satisfaction among non-participants also increased, but on a lower level than participants. Our results underline the importance to offer and facilitate a high participation in relocation process activities to obtain a satisfying relocation to ABW.

## Figures and Tables

**Table 1:** Descriptive data at baseline for employees participating and not participating in activities; sex, age, manager, office type, number of activities attended, and satisfaction with knowledge, office rules, information and support.

	Total *n* = 699		Participants *n* = 439		Non-participants *n* = 260	
	*n* (%)	Mean (SD)	*n* (%)	Mean (SD)	*n* (%)	Mean (SD)
Women	312 (45)		221 (50)		91 (35)	
Men	384 (55)		216 (50)		168 (65)	
Managers	59 (8)		41 (9)		18 (7)	
Age		45.6 (10.5)		46.2 (10.3)		45.1 (10.6)
Highsatisfaction psychosocial work environment	500 (78)		340 (77)		160 (62)	
Office type						
Private office	257 (37)		189 (43)		68 (26)	
Sharedroom/open-plan office	442 (63)		250 (57)		192 (74)	
Participation in activities						
0 activities	260 (37)				260 (37)	
1 activity	171 (25)		171 (39)			
2 activities	148 (21)		148 (34)			
3 activities	76 (11)		76 (17)			
4 activities	44 (6)		44 (10)			
High satisfaction						
Knowledge		56 (30)		65 (25)		40 (30)
Office rules		58 (28)		67 (23)		43 (30)
Information		54 (28)		63 (25)		41 (28)
Support		59 (30)		68 (26)		44 (32)

**Table 2:** Changes in satisfaction with knowledge, office rules, information and support three and nine months after relocation compared with before relocation. Estimates (B) indicate the change compared with before relocation (*n* = 696–699).

	Unadjusted	Adjusted
**Knowledge**	B (95% CI)	B (95% CI)
Intercept	40.4 (37.0–43.7) **	51.5 (38.9–64.0) **
3 months after	14.7 (10.4–18.9) **	9.7 (−4.5–24.0)
9 months after	16.4 (12.2–20.7) **	18.0 (3.4–32.7) *
**Office rules**		
Intercept	42.9 (39.8–47.0) **	40.0 (27.7–51.3) **
3 months after	23.1 (19.3–27.0) **	26.4 (13.4–39.5) **
9 months after	20.7 16.5–24.8) **	21.1 (7.0–35.3) **
**Information**		
Intercept	40.6 (37.3–44.0) **	44.0 (31.3–56.7) **
3 months after	7.3 (2.4–12.3) **	8.4 (−8.0–24.7)
9 months after	6.9 (1.7–12.1) *	29.3 (11.6–46.9) **
**Support**		
Intercept	43.7 (40.1–47.2) **	39.8 (26.6–53.0) **
3 months after	13.4 (8.6–18.2) **	12.4 (−3.9–28.7)
9 months after	12.0 (6.8–17.1) **	7.7 (−9.9–25.2)

* *p* < 0.05. ** *p* < 0.001. Adjusted for age, sex and satisfaction with psychosocial work environment.

**Table 3:** Dose—response effects of participation in 1–4 activities on satisfaction with knowledge, office rules, information and support. Estimates (B) indicate the difference from non-participants (*n* = 696–699).

	Unadjusted	Adjusted
**Knowledge**	B (95% CI)	B (95% CI)
1 activity	17.2 (11.8–22.5)	15.7 (10.4–20.9)
2 activities	28.1 (22.6–33.7)	25.6 (20.0–31.1)
3 activities	27.5 (20.5–34.5)	25.0 (18.0–32.0)
4 activities	36.9 (28.1–45.7)	33.9 (25.1–42.7)
**Office rules**		
1 activity	17.2 (12.2–22.2)	15.1 (10.1–20.0)
2 activities	27.1 (21.8–32.3)	24.2 (18.9–29.4)
3 activities	28.9 (22.3–35.6)	26.0 (19.4–32.7)
4 activities	31.2 (22.9–39.5)	27.6 (19.3–35.9)
**Information**		
1 activity	16.5 (11.1–21.8)	15.0 (9.7–20.3)
2 activities	24.8 (19.2–30.4)	22.7 (17.1–28.3)
3 activities	24.9 (17.9–32.0)	22.8 (15.7–29.9)
4 activities	29.4 (20.6–38.2)	26.8 (17.8–35.7)
**Support**		
1 activity	17.8 (12.2–23.5)	15.00 (9.5–20.5)
2 activities	25.1 (19.2–31.0)	21.4 (15.6–27.2)
3 activities	34.2 (26.7–41.7)	29.8 (22.4–37.2)
4 activities	32.2 (22.9–41.6)	26.6 (17.3–35.8)

All outcomes *p* < 0.001. Adjusted for age, sex and satisfaction with psychosocial work environment.

**Table 4:** Dose—response effects of participation in process activities on changes in satisfaction with knowledge, office rules, information and support three and nine months after relocation. Estimates (B) show the change in satisfaction in the participation groups compared with the change in the non-participation group from baseline to three and nine months after relocation. *n* = 696–699.

	3 Months Follow-Up	9 Months Follow-Up
**Knowledge**	B (95% CI)	B (95% CI)
Activities 1	−4.16 (−10.10–1.77)	−7.42 (−13.49–−1.36) *
Activities 2	−9.66 (−15.88–−3.45) *	−11.73 (−18.06–−5.40) **
Activities 3	−8.73 (−16.15–−1.30) *	−10.80 (−18.43–−3.18) *
Activities 4	−17.73 (−26.89–−8.57) **	−17.12 (−26.41–−7.83) **
**Office rules**		
Activities 1	−6.55 (−11.99–−1.11) *	−6.87 (−12.74–−1.00) *
Activities 2	−14.16 (−19.85–−8.46) **	−11.94 (−18.07–−5.81) **
Activities 3	−11.57 (−18.43–−4.72) **	−17.77 (−25.18–−10.36) **
Activities 4	−13.81 (−22.29–−5.33) **	−16.00 (−25.06–−6.95) *
**Information**		
Activities 1	−8.91 (−15.72–−2.09) *	−6.87 (−14.17–0.43)
Activities 2	−6.46 (−13.59–0.68)	−5.02 (−12.63–2.59)
Activities 3	-6.64 (−15.13–1.85)	−5.98 (−15.12–3.16)
Activities 4	−8.66 (−19.12–1.79)	−6.00 (−17.09–5.08)
**Support**		
Activities 1	−6.57 (−13.37–0.23)	−6.01 (−13.27–1.25)
Activities 2	−8.68 (−15.80–−1.56) *	−11.49 (−19.07–−3.92) **
Activities 3	−18.05 (−26.56–−9.53) **	−17.83 (−26.96–−8.70) **
Activities 4	−18.08 (−28.59–−7.58) **	−19.50 (−30.61–−8.39) **

* *p* < 0.05, ** *p* < 0.001. Model adjusted for age, sex and satisfaction with psychosocial work environment.

## Data Availability

Data are available upon reasonable request.
